# *Bacillus licheniformis* Reshapes the Gut Microbiota to Alleviate the Subhealth

**DOI:** 10.3390/nu14081642

**Published:** 2022-04-14

**Authors:** Siyuan Feng, Chen Meng, Zikai Hao, Hong Liu

**Affiliations:** 1 Beijing Advanced Innovation Center for Biomedical Engineering, Beihang University, Beijing 100083, China; feng123204@163.com (S.F.); mmenger@126.com (C.M.); 2 Institute of Environmental Biology and Life Support Technology, School of Biological Science and Medical Engineering, Beihang University, Beijing 100083, China; 3 State Key Laboratory of Software Development Environment, School of Computer Science and Engineering, Beihang University, Beijing 100083, China; 4 International Joint Research Center of Aerospace Biotechnology & Medical Engineering, Beihang University, Beijing 100083, China

**Keywords:** *Bacillus licheniformis*, subhealth, gut microbiota, antibiotics

## Abstract

Subhealth is a condition between health and disease that has become a common public health risk. Therefore, it is necessary to find more scientific therapies that can alleviate the symptoms of subhealth effectively. The gut microbiota is closely associated with subhealth. As a mature probiotic preparation, *Bacillus licheniformis* (*B. licheniformis*) can regulate gut microbiota balance, which indicates that *B. licheniformis* has the potential in regulating subhealth. This study produced the subhealthy rats by using chronic stress for 4 weeks to simulate psychological stress, with excessive antibiotics for 1 week to simulate bad living habits. Then, they were treated for 4 weeks with *B. licheniformis*. The results showed that *B. licheniformis* could recover the gut microbiota balance that had been destroyed by subhealth. The serum corticosterone and the proinflammatory cytokine tumor necrosis factor-α decreased after being treated by *B. licheniformis*. *B. licheniformis* also reduced glutamic acid and norepinephrine levels while increasing γ-aminobutyric acid and 5-hydroxytryptamine levels in the brain. In addition to the physiological changes, *B. licheniformis* decreased the anxiety-like behaviors of rats. Therefore *B. licheniformis* could alleviate the subhealth state, mainly by remodeling the gut microbiota, reducing inflammation, inhibiting the hypothalamic–pituitary–adrenal axis hyperactivity, regulating neurotransmitter levels, and easing a negative mood.

## 1. Introduction

Subhealth refers to the state between health and disease and is characterized as the disorder of psychological behaviors or physiological characteristics or some physical examination indices without typical pathological characteristics [1]. The most common factors causing a subhealth state are psychological stress, bad habits, lack of relaxation and physical exercise, long working hours, and air and noise pollution [2], including chronic stress and antibiotic abuse. Subhealth has become a common public health risk. Therefore, there is an increasing need for scientifically validated therapies that can effectively alleviate subhealth symptoms.

More and more studies have found that gut microbiota is closely associated with diseases and health [3]. The gut microbiota consists of a group of microbes (mainly including bacteria) in the host’s intestinal tract, with the number of bacteria at nearly 100 trillion [4]. These bacteria participate in many physiological and biochemical reactions of the host, such as immune regulation [5], short-chain fatty acids (SCFAs) metabolism [6], and vitamin synthesis [7], by helping to decompose complex polysaccharides or stimulating the intestinal wall directly. They are crucial to the host’s immune system development, digestion function, and metabolism. Gut microbial composition imbalance and the corresponding changes in its function are also associated with inflammatory bowel disease, antibiotic-associated diarrhea, neurological disorders, and other clinical diseases [8]. With the deepening of research, it is gradually found that gut microbiota is associated with not only diseases but also with subhealth. For example, some studies showed that the traditional Asian diet could contribute to beneficial metabolic effects by optimizing gut microbiota and alleviating the subhealth status [9].

*Bacillus licheniformis* (*B. licheniformis*) is a Gram-positive bacillus, facultatively anaerobic bacteria. It is one of the dominant populations of the soil and plant microbiota but is not an inherent bacterium in the human intestinal tract [10]. *B. licheniformis* live bacterial preparation (Zhengchangsheng^®^, Northern Pharmaceutical Group Shenyang No.1 Pharmaceutical Co., Ltd., Shenyang, China) is a new microecological preparation made from the *B. licheniformis* BL20386 strain. The strain was isolated and cultured from a vaginal swab of a healthy pregnant woman in 1986 by Wu Tielin and then identified as *B. licheniformis* 20386 by the Institute of Microbiology of the Chinese Academy of Sciences [10]. It can regulate gut microbiota balance and is mainly used for acute and chronic diarrhea, ulcerative colitis, spontaneous bacterial peritonitis, and other intestinal diseases caused by the gut microbiota imbalance and other reasons in clinical practice. Many clinical cases can prove its safety and clinical effectiveness [11,12].

With the expansion of the application range of *B. licheniformis* in recent years, it is used to treat not only diarrhea and intestinal inflammation but also other diseases caused by gut microbiota disorder. Many clinical studies have found that it affects the impact on the patients’ inflammatory response [13,14], neurotransmitter levels [15], etc. Previous studies on *B. licheniformis* were mostly associated with diseases, and its beneficial effect on improving health, especially the alleviation of subhealth status, has not been studied.

Therefore, this study used chronic stress to simulate psychological stress and excessive antibiotics to simulate bad living habits to leave rats in a subhealth state. *B. licheniformis* was used to explore whether its regulatory effect on gut microbiota could help improve health.

## 2. Materials and Methods

### 2.1. Experience Design

This study was approved by the Biological and Medical Ethics Committee of Beihang University. Animals were treated according to the National Institute of Health Guide for the Care and Use of Laboratory Animals. In this study, 68 Sprague Dawley 5- to 6-week-old male rats (specific pathogen-free), weighing 150 g to 170 g, were obtained from SiPeiFu (Beijing, China). A total of 4 to 5 rats were housed per cage and kept on a 12 h light/dark cycle, a temperature of 25 °C, and a humidity between 40% and 70%. The body weight was measured, the food intake was counted, and feces were collected once weekly.

**Figure 1:**
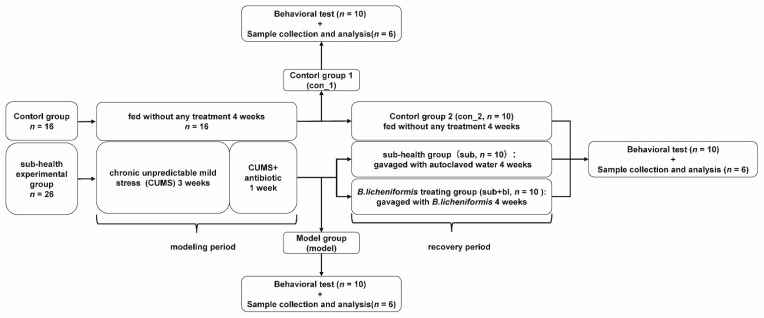
Experimental procedures.

The experiment period lasted for 8 weeks. The first 4 weeks (week 1–4) were the modeling period, during which rats were divided into 2 groups randomly after 1 week of adaptive feeding (week 0): the control group (*n* = 16) was fed without any treatment, at the end of the fourth week, 6 rats were randomly selected for sample collection (con_1, *n* = 6) and 10 rats were randomly selected for behavior test (con_1, *n* = 10); the subhealth model group (*n* = 26) was treated with chronic unpredictable mild stress (CUMS, week 1–4) and antibiotics (week 3 and 4), at the end of the fourth week, 6 rats were randomly selected for sample collection (model, *n* = 6) and 10 rats were randomly selected for behavior test (model, *n* = 10). The second 4 weeks (week 5–8) were the recovery period, during which rats in the subhealth model group were randomly divided into two groups: the subhealth group (sub, *n* = 10) was given sterile water by gavage for 4 weeks; the *B. licheniformis* treating group (sub+bl, *n* = 10) was treated by *B. licheniformis* gavage for 4 weeks; and the remaining rats in the control group were fed without any treatment (con_2, *n* = 10). At the end of the eighth week, all rats in each group were tested for behavior, and six rats were selected to kill randomly for sample collection. The sample collection included the brain, serum, intestinal contents, adrenal gland, spleen, and liver. The samples were stored at −80 °C before detection (Figure 1).

### 2.2. Subhealthy Rat Model

In this study, CUMS was used to simulate psychological stress, and excessive antibiotic treatment was used to simulate bad living habits. Among them, the CUMS process was a modified version of that described previously [16]. Rats were randomly exposed to 2 of 7 stressors per day for 4 weeks, and the stressors for 2 consecutive days were completely different. The stressors included: (1) food deprivation for 24 h, (2) water deprivation for 24 h, (3) day and night reversed for 24 h, (4) shake at 170 rpm for 1 h, (5) oven 50 °C for 10 min, (6) swim in ice water for 5 min, and (7) tail pinch for 1 min. The CUMS process is shown in Table S1 (Supplement Materials).

In the fourth week, the model group was treated with mixed antibiotics. According to the method described by Reikvam et al. [17], rats were gavaged with a solution of 35 mg/kg vancomycin hydrochloride (Sigma-Aldrich Corp., St. Louis, MO, USA), 70 mg/kg neomycin sulfate (Amresco Inc., Framingham, MA, USA), and 70 mg/kg metronidazole (Macklin, China) once every 12 h for 7 days. The mixed antibiotic solution was prepared daily for use. Ampicillin sodium (Amresco, Inc., Framingham, MA, USA) was added to the drinking water (1 mg/mL), and rats were provided *ad libitum* with drinking water renewed daily.

### 2.3. Preparation of B. licheniformis Suspension and Gavage

*B. licheniformis* was prepared into a 1 × 10^8^ CFU/mL bacterial suspension in sterile water. For 5 to 8 weeks, the sub+bl group was given *B. licheniformis* by gavage at 1 mL, whereas the subgroup was given 1 mL sterile water by gavage once daily for 28 days.

### 2.4. Behavioral Testing

#### 2.4.1. Forced Swimming Test (FST) 

Ten rats in each group were randomly selected for an FST to measure the depression-like behaviors and were put back in cages after the test. At the beginning of the test, a rat was placed into a limited space (700 mm high, 400 mm in diameter) with enough water (170–300 mm deep). The immobility time and immobility count of rats in 5 min were recorded and analyzed by the animal behavior test and analysis system (Labmaze V3.0, Beijing, China). After the test, the rat was taken out and dried. Water was renewed between different treatment groups [16,18].

#### 2.4.2. Elevated plus Maze (EPM) Test

Ten rats in each group were randomly selected for the EPM test to measure the anxiety-like behaviors and were put back in cages after the test. The test was carried out in a plus-maze device (1 m high) consisting of two cross open arms (425 × 120 mm) and two cross closed arms (425 × 120 × 225 mm). At the beginning of the test, a rat was placed in the central area of the maze facing an open arm, and the activity of the rat within 5 min was recorded and analyzed by the animal behavior test and analysis system (Labmaze V3.0, Beijing, China). After the test, the arms were cleaned with alcohol to remove the odor of the previous rat [16,19].

### 2.5. Measurement of Cytokines and Neurotransmitters

Six rats were randomly selected from each group for sample collection. Serum samples were collected from femoral vascular for the measurement of cytokines and corticosterone (CORT). Brain samples were collected for the measurement of neurotransmitters. Enzyme-linked immunosorbent assay kits were used to measure CORT, interferon-γ (IFN-γ), interleukin-6 (IL-6), and tumor necrosis factor-α (TNF-α) in the serum, and 5-hydroxytryptophan (5-HT) and dopamine (DA) in the whole brain.

Neurotransmitters (including tryptophan (Trp), tyrosine (Tyr), glutamic acid (Glu), glutamine (Glu), γ-aminobutyric acid (GABA), epinephrine, norepinephrine (NE), 5-HT, and 5-hydroxyindole-3-acetic acid (5-HIAA)) in the whole brain were measured by liquid chromatography (LC) -mass spectrometry (MS). The brain was weighed and added with methanol solution (containing 10% formic acid) and double-distilled water (1:1, *v*/*v*) and then vibrated at 60 Hz for 1 min and repeated twice. After centrifugation at 4 °C for 5 min at 12,000 rpm, the supernatant was added with 100 ppb dual-isotope internal standard at 1:1. Vortex oscillation was performed for 30 s. The supernatant was filtered through a 0.22 μm membrane.

LC conditions: Samples were analyzed at 40 °C by high-performance LC on an Acquity™ UPLC BEH C18 column (2.1 × 100 mm, 1.7 μm; Waters). The injection volume was 5 μL. Mobile phases consisted of (A) 10% methanol-water (containing 0.1% formic acid) and (B) 50% methanol-water (containing 0.1% formic acid). Solvent B increased from 20% to 100% between 0 and 1 min, solvent B remained at 100% between 1 and 7 min, solvent B was reduced from 100% to 20% between 7 and 7.5 min, and solvent B remained at 20% between 7.5 and 11 min. The flow rate was set as 0.4 mL/min. 

MS conditions: electrospray ionization source, positive-ion ionization mode. The temperature of the ion source was set as 500 °C. The ion source voltage was 5500 V. Multiple response monitoring was used for scanning.

### 2.6. Measurement of SCFAs

Six rats were randomly selected from each group to be killed and collect the colon contents. The colon contents were added with 15% phosphoric acid, 125 μg/mL isohexanoic acid, and diethyl ether. The samples were homogenized for 1 min and centrifuged at 4 °C at 12,000 rpm for 10 min. Gas chromatography (GC)-MS was used to detect SCFAs in the supernatant.

GC conditions: HP-INNOWax capillary column (30 m × 0.25 mm ID × 0.25 μm; Agilent Technologies Inc., Santa Clara, CA, USA) was used to separate SCFAs. The injection volume was 1 μL in a 10:1 split mode, the injection temperature was 250 °C, the ion source temperature was 230 °C, the transfer line temperature was 250 °C, and the quadrupole temperature was 150 °C. The temperature gradient increased at 10 °C/min from 90 °C to 120 °C, increased at 5 °C/min from 120 °C to 150 °C, increased at 25 °C/min from 150 °C to 250 °C, and held for 2 min at 250 °C. The carrier gas was helium with a flow rate of 1.0 mL/min. MS conditions: electron impact ionization source, SIM scanning mode, electron energy of 70 eV.

### 2.7. 16S rDNA Sequencing and Analysis 

The total genomic DNA of the microbiota in feces was extracted by the CTAB method. DNA was amplified by polymerase chain reaction (PCR) with a pair of specific primers 341F (CCTAYGGRBGCASCAG)/806R (GGACTACNNGGGTATCTAAT) with barcode. The PCR products were purified using 2% agarose gel electrophoresis, and the target bands were recovered. The multiple amplified library of the V3 + V4 region of the 16S rDNA gene was constructed. The library was quantitated by qubit, qualified by library detection, and sequenced by the NovaSeq6000 sequencing system.

Raw 16S rDNA sequencing data were analyzed by R (version 3.6.1), and operational taxonomic unit (OTU) sequences were queried against the Silva taxonomic annotation database to obtain taxonomic information. The community composition of each sample was calculated at each taxonomic level (phylum, class, order, family, genus, and species). The richness, Chao1, Shannon, and Simpson indices were calculated at the 97% similarity level for α-diversity analysis. Based on OTU, an evolutionary tree was constructed to generate a distance matrix for β-diversity analysis.

### 2.8. Statistical Analysis

Statistical analyses and plots were performed in R software (version 3.6.1, https://www.r-project.org/, accessed on 1 August 2021). Wilcoxon test was used to analyze the significance of each index between different groups and find out the indices with a significant difference. Linear discriminant analysis (LDA) effect size (LEfSe) was used to find the microbial markers with a statistical difference, which means that LDA score > 2 and *p* < 0.05 (http://huttenhower.sph.harvard.edu/galaxy/, accessed on 5 August 2021).

## 3. Results

### 3.1. Changes in Body Weight, Food Intake, and Organ Indices

**Figure 2:**
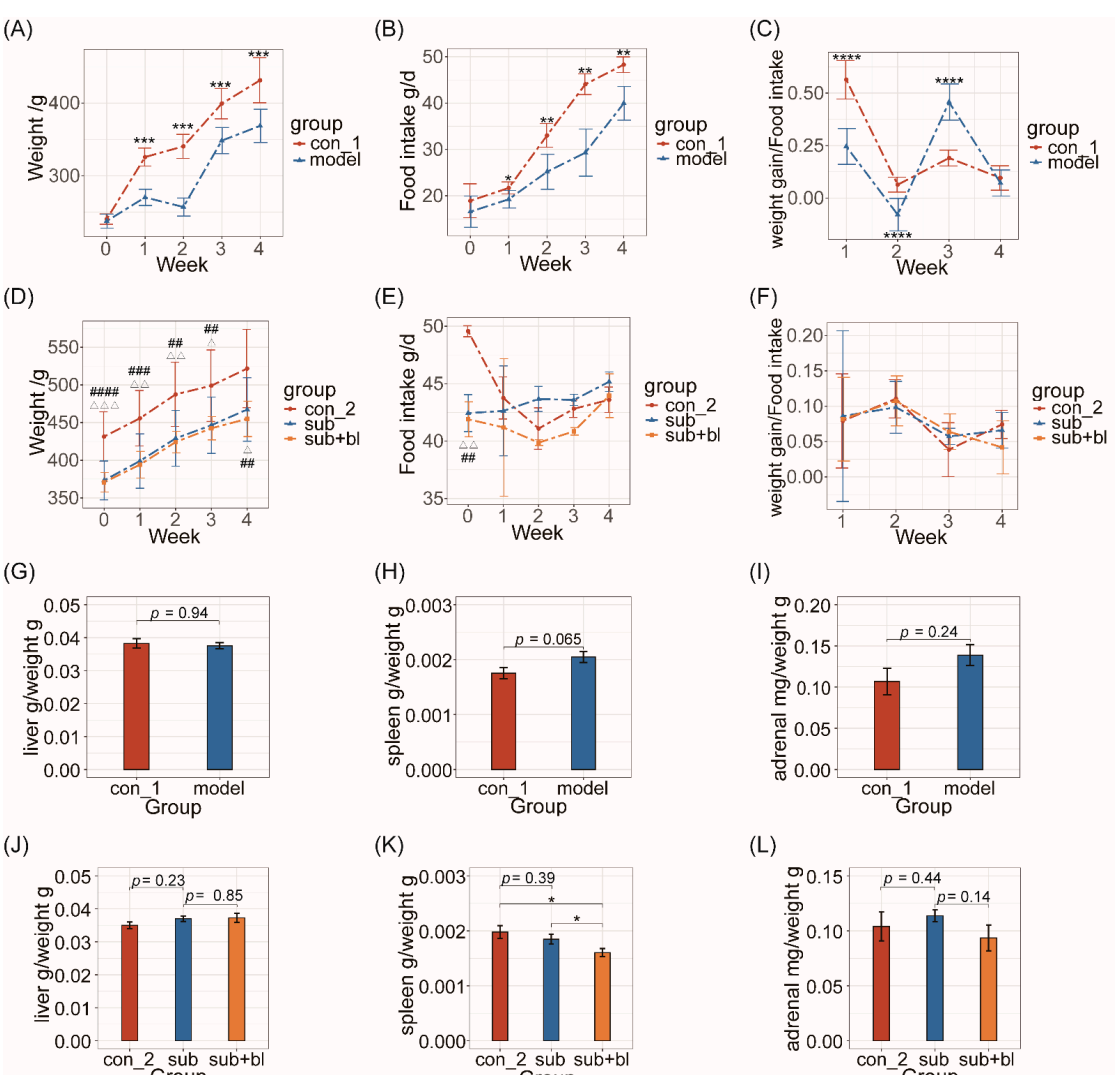
Effect of simulated subhealth on body weight, food intake, liver index, spleen index, and adrenal index (**A**) Body weight in the modeling period (**B**) Food intake in the modeling period (**C**) Weight gain/food intake ratio in the modeling period. Week 0: the end of adaptation; week 1–4: the end of each week in modeling period. * *p* < 0.05; ** *p* < 0.01; *** *p* < 0.001; **** *p* < 0.0001. (**D**) Body weight in the recovery period (**E**) Food intake in the recovery period (**F**) Weight gain/food intake ratio in the recovery period. Week 0: the end of modeling period; week 1–4: the end of each week in recovery period. con_2 vs. sub ^△^ *p* < 0.05, ^△△^ *p* < 0.01, ^△△△^ *p* < 0.001; con_2 vs. sub+bl ^##^ *p* < 0.01, ^###^ *p* < 0.001, ^####^ *p* < 0.0001. (**G**) Liver index: liver/body weight ratio at the end of the modeling period (**H**) Spleen index: spleen/body weight ratio at the end of the modeling period (**I**) Adrenal index: adrenal/body weight ratio at the end of the modeling period (**J**) Liver index: liver/body weight ratio at the end of the recovery period (**K**) Spleen index: spleen/body weight ratio at the end of the recovery period (**L**) Adrenal index: adrenal/body weight ratio at the end of the recovery period. * *p* < 0.05.

The body weight and food intake of the model group were significantly lower than those of the con group from the first weekend of subhealth treatment (*p* < 0.05; Figure 2A,B), and a negative weight growth was found at the end of the second week. The weight gain/food intake ratio of the model group was significantly lower than the con group in the first 2 weeks (*p* < 0.0001; Figure 2C) and significantly higher than the con group in the third week (*p* < 0.0001; Figure 2C). After 4 weeks of subhealth treatment, the spleen index of the model group increased significantly (*p* = 0.065; Figure 2H), and the adrenal/body weight ratio increased (Figure 2I), but the liver index had no significant difference (Figure 2G).

After 4 weeks of the recovery period, the body weight of the subgroup was still significantly lower than the con group (*p* < 0.05; Figure 2D), but there was no significant difference between the sub+bl and subgroups, suggesting that *B. licheniformis* did not affect the body weight.

The spleen index of the subgroup returned to the same level as the con group after the recovery period, whereas the spleen index of the sub+bl group was significantly lower than the other groups (*p* < 0.05; Figure 2K). There was no significant difference in the adrenal index between the con and subgroups, but the adrenal index of the sub+bl group was lower than the subgroup (*p* = 0.14; Figure 2L). At the same time, there was no significant difference in liver index among the groups (Figure 2J). These results suggested that *B. licheniformis* can reduce the adrenal gland/body weight ratio and spleen index but not the liver index.

### 3.2. Behavioral Changes

**Figure 3:**
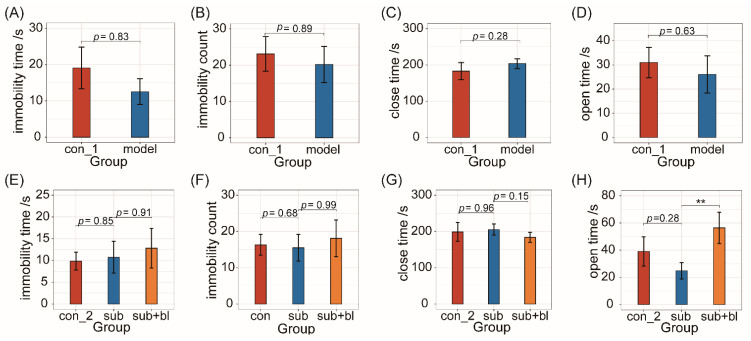
*B. licheniformis* reduced anxiety-like behaviors. (**A**,**B**) Immobility time and immobility count in FST at the end of the modeling period. (**C**,**D**) Time spent in the closed-arms and the open-arms at the end of the modeling period. (**E**,**F**) Immobility time and immobility count in FST at the end of the recovery period. (**G**,**H**) Time spent in the closed-arms and the open-arms at the end of the recovery period. ** *p* < 0.01.

In addition to physiological effects, chronic stress events and antibiotic treatment may also affect mental health. At the end of the fourth week, in the FST, there was no significant difference in immobility time and count between the model and con groups (Figure 3A,B). The behavioral tests showed that, in the EPM test, there was also no significant difference in the time spent in the closed and open arms between the con and model groups (Figure 3C,D). These results suggested no significant change in anxiety- and depression-like behaviors, although 5-HT levels in the brain decreased.

After treatment with *B. licheniformis* for 4 weeks, there was no significant difference in immobility time and immobility count among the 3 groups (Figure 3E,F), indicating that treatment with *B. licheniformis* had no significant effect on depression-like behaviors. In the EPM test, compared to the subgroup, the time in the closed arms of the sub+bl group decreased (*p* = 0.15; Figure 3G), and the time in the open arms of the sub+bl group increased significantly (*p* < 0.01; Figure 3H), indicating that *B. licheniformis* could reduce the host’s anxiety-like behaviors.

### 3.3. Changes in Cytokines and Nervous System-Related Metabolites

**Figure 4:**
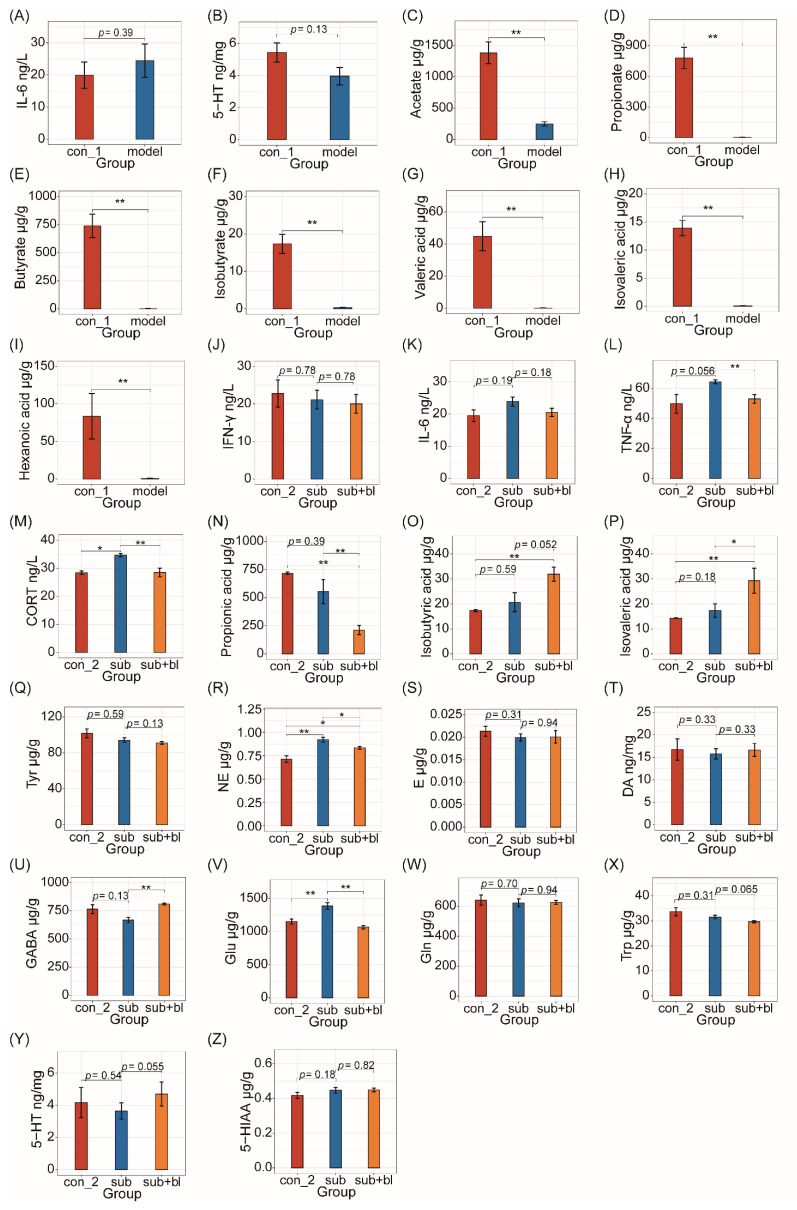
Changes in cytokines and nervous system-related metabolites (**A**) IL-6 levels in serum at the end of the modeling period (**B**) 5-HT levels in the brain at the end of the modeling period (**C**–**I**) Acetic acid, propionic acid, butyric acid, isobutyric acid, valeric acid, isovaleric acid, and hexanoic acid levels in the colon at the end of the modeling period (**J**) IFN-γ levels in serum at the end of the recovery period (**K**) IL-6 levels in serum at the end of the recovery period (**L**) TNF-α levels in serum at the end of the recovery period (**M**) CORT levels in serum at the end of the recovery period (**N**) Propionic acid levels in colon contents at the end of the recovery period (**O**) Isobutyric acid levels in colon contents (**P**) Isovaleric acid levels in colon contents at the end of the recovery period (**Q**) Tyr levels in the brain at the end of the recovery period (**R**) Levels of NE in the brain at the end of the recovery period (**S**) Levels of E in the brain at the end of the recovery period (**T**) DA levels in the brain at the end of the recovery period (**U**) GABA levels in the brain at the end of the recovery period (**V**) Glu levels in the brain (**W**) Gln levels in the brain at the end of the recovery period (**X**) Trp levels in the brain at the end of the recovery period (**Y**) 5-HT levels in the brain at the end of the recovery period (**Z**) 5-HIAA levels in the brain at the end of the recovery period. * *p* < 0.05; ** *p* < 0.01.

After 4 weeks of subhealth treatment, the IL-6 levels of the model group increased (*p* = 0.39; Figure 4A). The other cytokines (TNF-α and IFN-γ) had no significant difference between the groups. Neurotransmitters in the brain had no significant difference, except that the 5-HT levels increased in the model group (*p* = 0.13; Figure 4B). SCFAs, a common intestinal metabolite, in the colon contents decreased significantly after subhealth modeling (*p* < 0.01; Figure 4C–I).

To explore the effects of *B. licheniformis* on the inflammatory response, three inflammatory cytokines (TNF-α, IFN-γ, and IL-6) in the serum were detected. Among them, the IFN-γ levels had no significant difference among the three groups (Figure 4J). After 4 weeks, the IL-6 levels still increased in the subgroup (*p* = 0.19; Figure 4K) but decreased in the sub+bl group (*p* = 0.18; Figure 4K). Although TNF-α in the model group had no significant difference from the con group after the modeling period, TNF-α in the subgroup was significantly higher than in the con group (*p* = 0.056; Figure 4L). TNF-α in the sub+bl group significantly decreased (*p* < 0.05; Figure 4L).

To explore the effects of *B. licheniformis* on the hypothalamic–pituitary–adrenal (HPA) axis, CORT in the serum was measured. There was no significant difference in the serum CORT between each group on the fourth week, but the CORT of the subgroup was significantly higher than the con group during the modeling period (*p* < 0.05; Figure 4M). After treatment with *B. licheniformis*, the serum CORT of the sub+bl group was significantly lower than the subgroup (*p* < 0.05; Figure 4M). The propionic acid level in the sub+bl group was significantly lower than the other groups (*p* < 0.01; Figure 4N), whereas the isobutyric acid level (*p* = 0.052) and isovaleric acid level (*p* < 0.05) significantly increased (Figure 4O,P).

To further explore the reason why *B. licheniformis* can reduce anxiety-like behaviors, neurotransmitters in the brain were tested (Figure 4Q–Z). First, there was no significant difference in neurotransmitters, except for 5-HT, after the modeling period. However, after 4 weeks of the recovery period, GABA of the subgroup decreased compared to the con group (*p* = 0.13; Figure 4U), and Glu and NE significantly increased (*p* < 0.01; Figure 4V,R). Compared to the subgroup, 5-HT in the sub+bl group significantly increased (*p* = 0.055; Figure 4Y) and Trp significantly decreased (*p* = 0.065; Figure 4X), indicating that *B. licheniformis* might promote 5-HT synthesis in the brain. GABA in the sub+bl group was significantly higher than the subgroup (*p* < 0.01; Figure 4U), and Glu was significantly lower (*p* < 0.01; Figure 4V). NE in the sub+bl group significantly decreased (*p* < 0.05; Figure 4R), but its precursor Tyr did not change significantly (Figure 4Q), suggesting that the NE decrease may be due to the decreased synthesis or increased consumption of NE by the gut microbiota.

### 3.4. Changes in the Gut Microbiota

**Figure 5:**
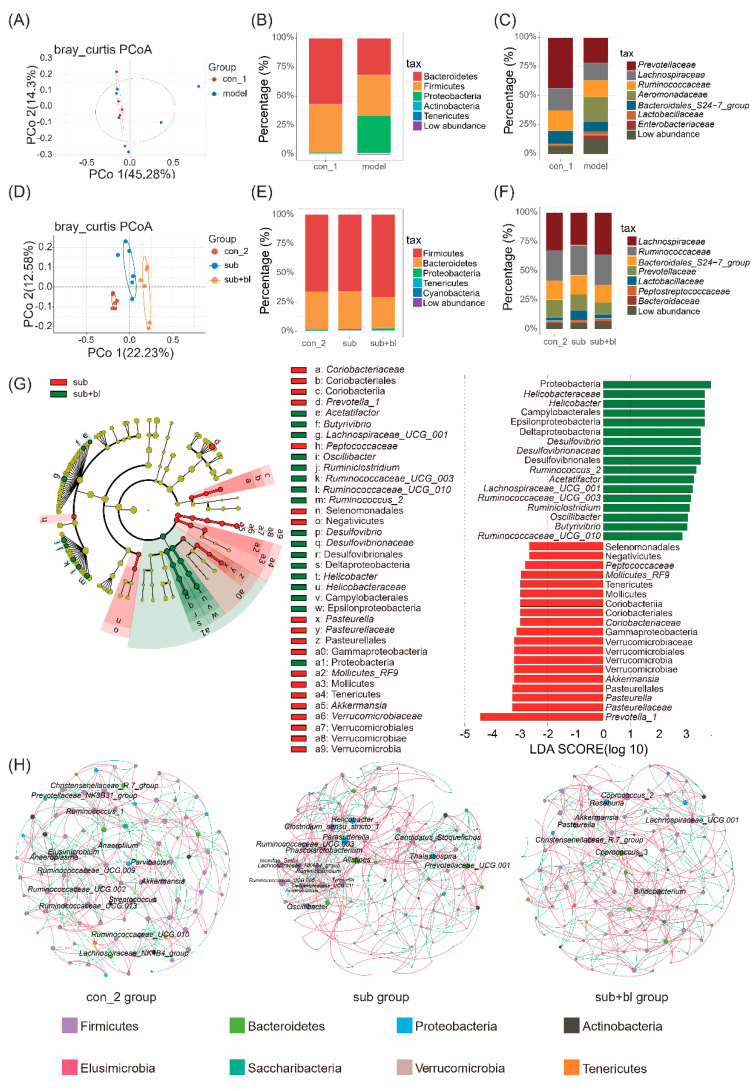
Changes in the gut microbiota. (**A**) PCoA of the gut microbiota at the end of the modeling period (**B**) Composition of the gut microbiota at the phylum level at the end of the modeling period (**C**) Composition of gut microbiota at the family level at the end of the modeling period (**D**) PCoA of gut microbiota at the end of the recovery period (**E**) Composition of the gut microbiota at phylum taxonomic ranking at the end of the recovery period (**F**) Composition of gut microbiota at family taxonomic ranking at the end of the recovery period (**G**) LEfSe for comparisons of the subgroup to the sub+bl group: taxonomic tree and effect size histograms (**H**) Microbial network at the end of the recovery period: the genus with degree >10 are marked. Different colors of the nodes represent different phylum. The red curve shows a positive correlation, and the green curve shows a negative correlation.

At the end of the fourth week, there was no significant difference in the α-diversity of the gut microbiota (Figure S1A–D, Supplement Materials). β-Diversity showed that the gut microbiota of the model and con groups changed but not significantly (*p* = 0.073, Adonis analysis; Figure 5A). After 4 weeks of subhealth simulation, the relative abundance of Proteobacteria increased, and that of Bacteroides decreased (Figure 5B). The relative abundance of *Prevotellaceae* decreased, whereas the relative abundance of potentially pathogenic *Enterobacteriaceae* and *Aeromonadaceae* increased (Figure 5C). It is worth noting that diarrhea was also observed in the model group.

Because *B. licheniformis* can regulate the gut microbiota, this study explored the effects of *B. licheniformis* on the gut microbiota. Principal coordinate analysis (PCoA) showed that the gut microbiota in each group was significantly separated (*p* < 0.01; Figure 5D). Although the recovery period lasted for 4 weeks, there was a significant difference between the gut microbiota in the sub and con groups (*p* = 0.002, Adonis analysis), and the gut microbiota in the sub+bl group was also significantly different from the subgroup (*p* = 0.0018, Adonis analysis).

At the level of α-diversity, there was no significant difference in the Richness index and Chao1 index of gut microbiota between the subgroup and the con group. The Richness index and Chao1 index in the sub+bl group were slightly higher than in the subgroup, but there was no significant difference (Figure S1E,F, Supplement Materials). The Shannon index of the sub+bl group was higher than the subgroup, indicating that the species diversity of the gut microbiota in the sub+bl group increased (Figure S1G,H, Supplement Materials).

After the recovery period, the differences in the phylum taxonomic ranking among the groups are shown in Figure 5E. The relative abundance of Firmicutes increased in each group over time. The differences in the family taxonomic ranking among the groups are shown in Figure 5F. The gut microbiota composition of the subgroup was still significantly different from the con group after the recovery period. The composition of the gut microbiota in the sub+bl group was closer to the con group, whereas the difference compared to that in the subgroup (Figure 5F) was significant, including the relative abundance of *Lachnospiraceae* increasing and the relative abundance of *Prevotellaceae* and *Lactobacillaceae* decreasing.

LEfSe showed that the relative abundance of Proteobacteria in the sub+bl group significantly increased (Figure 5G). A total of 13 genera had a significant change in relative abundance, with LDA scores > 2 (Figure 5G). In the sub+bl group, the relative abundance of *Acetatifactor*, *Butyrivibrio*, *Lachnospiraceae_UCG_001*, *Ruminococcaceae_UCG_003*, *Ruminococcaceae_UCG_010*, *Ruminiclostridium*, *Oscillibacter*, *Ruminococcus_2*, *Desulfovibrio*, and *Helicobacter* significantly increased. The relative abundance of *Prevotella_1, Pasteurella*, and *Akkermansia* significantly decreased (*p* < 0.05; Figure 5G).

To further explore the structural changes in the gut microbiota treated with *B. licheniformis*, Spearman’s correlation analysis was used to analyze the relationships among the top 100 genera with relative abundance. The parameters of each network are shown in Table S2 (Supplement Materials). The clustering coefficient of the gut microbiota network in the sub+bl group was lower than in the subgroup, indicating that the compactness of the network decreased, and the number of the genus with degrees >10 decreased from 16 to 8 (Figure 5F). The microbial network structure in the sub+bl group was closer to the con group. The central genera of the network are *Parasutterella* and *Clostridium_sensu_stricto_1* of the subgroup to *Coprococcus_2* of the sub+bl group, suggesting that *B. licheniformis* could help *Coprococcus_2* colonization and inhibit *Parasutterella* and *Clostridium_sensu_stricto_1* colonization. *B. licheniformis* increased the degree of *Lachnospiraceae_UCG_001*, *Akkermansia* and *Pasteurella* and decreased the degree of *Ruminococcaceae_UCG_003*, *Ruminiclostridium* and *Helicobacter*. There were significant differences in the relative abundance of these genera after *B. licheniformis* treatment. Among genera whose relative abundance did not change, *B. licheniformis* also increased the degree of *Roseburia*, *Bifidobacterium*, and *Coprococcus_3* and decreased the degree of *Ruminococcaceae_UCG_001* and *Prevotellaceae_UCG_001* (Figure 5F).

### 3.5. Correlation Analysis of the Gut Microbiota and Physiological or Behavioral Indices

**Figure 6:**
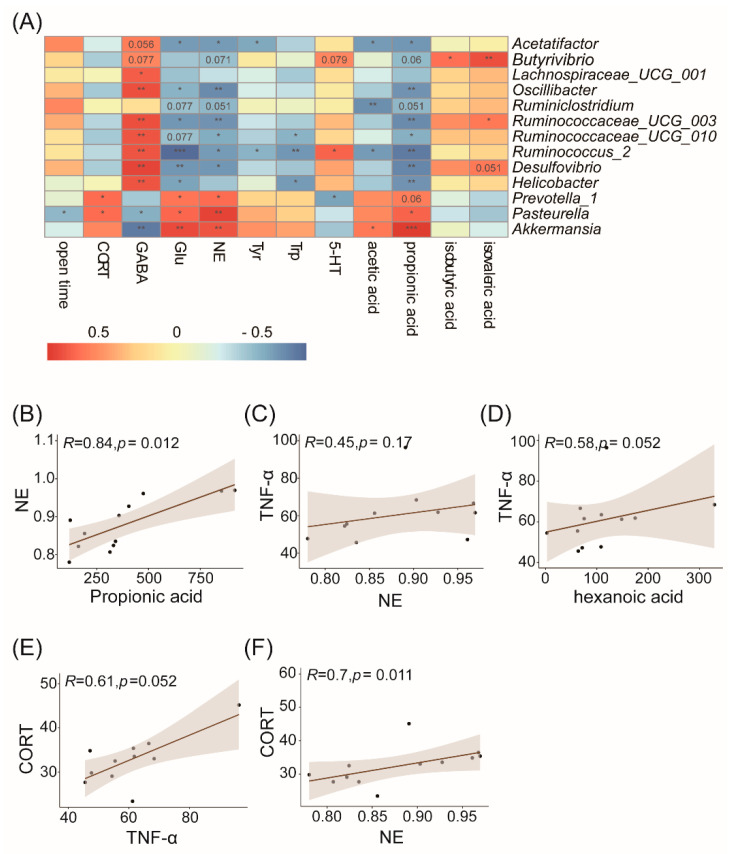
Correlation among relative abundance of the gut microbiota, and physiological and behavioral indices. (**A**) Correlation among the genera whose relative abundance changed significantly and the physiological or behavioral indices. The red cell means positive correlation, the blue cell means negative correlation, the value in the cell is the *p*-value, and the blank cell indicates that the correlation coefficient was not significant. * *p* < 0.05; ** *p* < 0.01; *** *p* < 0.001 (**B**) Propionic acid and NE (**C**) NE and TNF-α (**D**) Hexanoic acid and TNF-α (**E**) TNF-α and CORT (**F**) NE and CORT.

Spearman’s correlation analysis was conducted among genera whose relative abundance changed significantly and the physiological or behavioral indices in the sub and sub+bl group (Figure 6A). Almost all genera were significantly correlated with GABA and Glu, suggesting that the change in the gut microbiota played a role in the Gln/Glu-GABA cycle. Furthermore, these genera were also significantly correlated with propionic acid. In these genera, *Pasteurella* and *Prevotella_1*, which were significantly reduced in the sub+bl group, were positively correlated with NE in the brain and serum CORT, and the increased *Acetatifactor*, Oscillibacter, *Ruminiclostridium*, *Ruminococcaceae_UCG_010*, *Ruminococcaceae_UCG_003*, *Ruminococcus_2*, and *Desulfovibrio* were negatively correlated with NE in the brain, suggesting that the changes in the composition and structure of the gut microbiota had a significant effect on NE in the brain. Moreover, *Ruminoccus_2* was positively correlated with 5-HT levels in the brain, and *Prevotella_1* was negatively correlated with 5-HT levels in the brain. These results suggested that the change in the gut microbiota may be the reason why *B. licheniformis* had a beneficial effect on subhealth and reduced anxiety. *Pasteurella* was negatively correlated with the time in open arms, but the other genera were not significantly correlated with the behaviors. The results showed that *B. licheniformis* could reduce the anxiety of the sub+bl group not because of the relative abundance change of a certain genus but the changes in the gut microbiota compositional structure. The changed gut microbiota was correlated with the decreased NE levels and increased GABA and 5-HT levels in the brain and thus may reduce inflammation, HPA axis hyperactivity, and anxiety.

## 4. Discussion

This study first used antibiotic treatment and chronic stress to simulate the subhealth of rats. The results showed that the gut microbiota of rats after subhealth treatment was damaged. SCFAs in the colon, the main metabolites in the gut microbiota [20,21], decreased, proinflammatory cytokines in the serum increased, spleen index increased, and neurotransmitter levels in the brain decreased, as well as other physiological abnormalities. However, the rats did not present pathological behaviors. Moreover, some indicators (such as the cytokine TNF-α) did not significantly differ between the con and model groups at the end of the fourth week. However, after another 4 weeks, there was a significant difference between the con and subgroups (Figure 4L). These results showed that after experiencing adverse events, although some indicators reflecting the health status did not change significantly, these indicators showed significant worsening over time.

Treatment with *B. licheniformis* for 4 weeks produced many beneficial effects on subhealthy rats. First, *B. licheniformis* could reduce the adrenal index and reduce serum CORT. The adrenal gland is an important endocrine gland in the HPA axis. The relative adrenal size/weight is a useful biomarker of the general levels of the HPA axis, and increased adrenal weight is often observed in rats and mice exposed to various chronic stresses [22]. It is suggested that *B. licheniformis* help reduce HPA axis hyperactivity. At the same time, *B. licheniformis* could also promote the decrease in the spleen index. Previous studies showed that splenomegaly could be observed in intestinal inflammation [23]. Consistent with the decrease in spleen index, *B. licheniformis* also significantly reduce serum proinflammatory cytokine TNF-α, suggesting that *B. licheniformis* can help reduce inflammatory reactions in subhealthy rats. *B. licheniformis* changed the neurotransmitter levels in the brain, including increasing the beneficial neurotransmitters GABA and 5-HT [24,25] and reducing Glu and NE levels. It has been proposed that NE plays a very important role in anxiety caused by long-term stress and blocking its receptor can produce antianxiety effects [26,27]. *B. licheniformis* also reduce anxiety-like behaviors. These results show that *B. licheniformis* could decrease negative mood in rats from the aspects of psychology and behavior.

Second, *B. licheniformis* also changed the gut microbiota of subhealthy rats. The relative abundance of the 10 genera increased significantly, whereas that of the 3 genera decreased significantly. The genera with a significant increase in relative abundance are the beneficial *Ruminococcaceae* (*Ruminiclostridium* and *Ruminococcaceae_UCG_010*) and *Lachnospiraceae* (*Butyrivibrio*, *Lachnospiraceae_UCG_001*, etc.), which are more common in healthy people [28,29,30], whereas the genera with decreased relative abundance included *Pasteurella* [31], etc. In the network analysis, *Roseburia*, *Bifidobacterium*, and *Coprococcus_3* with an increased degree were also the genera with higher relative abundance in healthy people [32]. Studies showed that *B. licheniformis* can produce an anaerobic environment and secrete acetic acid and lactic acid [33], which may promote the colonization of the genera in the butyric acid-producing *Lachnospiraceae* family (such as *Roseburia* [34] and *Coprococcus* [35]) in the intestine. The relative abundance of the butyric acid-producing genera of the *Lachnospiraceae* family (*Butyivribrio* [36], *Lachnospiraceae_UCG_001* [37], etc.) increased after treatment with *B. licheniformis*, also beneficial to human health. Butyric acid has been confirmed to have beneficial effects in humans, mainly by affecting the brain’s immune environment and releasing serotonin and intestinal hormone in the intestinal nervous system, inhibiting histone deacetylase, and other aspects that affect brain function [38]. Moreover, it can improve host immunity [39], prevent colorectal tumors [40], and stimulate bone formation [41]. *Lachnospiraceae* is beneficial in many aspects. For example, one study found that there is more *Lachnospiraceae* family in nonobese individuals [42]. In another study, the relative abundance of the *Lachnospiraceae* family increased in the gut microbiota of depression patients who had treatment, and the relative abundance of some genera in *Lachnospiraceae* was positively correlated with brain-derived neurotrophic factor levels [43]. Increased *Lachnospiraceae* after treatment with *B. licheniformis* may be one reason why *B. licheniformis* alleviated the subhealth.

In this study, *B. licheniformis* increased isobutyric acid and isovaleric acid levels in the colon (Figure 4O,P). Isobutyric acid and isovaleric acid are branched SCFAs. Branched SCFAs can modulate the lipid and glucose metabolism in adipocytes, improving insulin sensitivity in individuals with metabolic disorders [44]. SCFAs have been shown to reduce inflammation [45]. In contrast, studies on branched-chain fatty acids also found that SCFAs may play a role in alleviating the inflammatory response, such as reducing the proinflammatory mediators IL-8 and nuclear factor-κB expression [46,47]. Isobutyrate can increase the total number of bacteria in the rumen of the animals [48]. Isovaleric acid can inhibit osteoclast differentiation to ameliorate osteoporosis [49] and relax colonic smooth muscles via the cyclic AMP/protein kinase A pathway [50]. It is worth noting that *B. licheniformis* preparation has been proven to treat diarrhea by many studies and has been used in the clinical treatment of diarrhea for many years [12,15]. Moreover, *B. licheniformis* significantly increased isovaleric acid in the colon in this study. It was hypothesized that relaxation of the colonic smooth muscle caused by isovaleric acid might be one mechanism why *B. licheniformis* relieve abdominal pain and diarrhea. Interestingly, in this study, *B. licheniformis* also increased the genera in the *Ruminococcaceae* family. The decreased relative abundance in the intestine was often accompanied by the increase in propionic acid levels, and the increased relative abundance was accompanied by the decrease in propionic acid levels. *Ruminococcaceae* can consume lactic acid and C2-C5 SCFAs [51]. Thus, it was hypothesized that the increase in the genera in the *Ruminococcaceae* family might reduce the propionic acid levels in the colon (Figure 4N).

Correlation analysis showed that the seven genera whose relative abundance increased significantly in the sub+bl group were negatively correlated with NE, whereas NE was significantly positively correlated with propionic acid (Figure 6B), also confirming that the decrease in propionic acid was associated with the decrease in NE levels. Previous studies showed that NE could stimulate bone marrow production and the release of myeloid cells and then promote proinflammatory cytokine TNF-α production [52]. However, there was no significant correlation between NE and TNF-α in this study (Figure 6C), suggesting another reason for the decrease in serum TNF-α in the sub+bl group. TNF-α had a significant positive correlation with hexanoic acid, although there was no significant difference in hexanoic acid levels between the sub and sub+bl groups (Figure 6D). Some studies also showed that hexanoic acid could promote inflammation [53], showing that the change in TNF-α may be associated with gut microbiota metabolism. Previous studies found that the proinflammatory cytokine is a powerful activator of the HPA axis, and the reduction in the proinflammatory cytokine can also reduce CORT release [54,55]. This study also found that TNF-α in the serum had a significant positive correlation with CORT in the serum (Figure 6E). *Prevotella_1* and *Pasteurella*, whose relative abundance decreased in the sub+bl group, were positively correlated with CORT, suggesting that the decrease in serum CORT may be associated with the gut microbiota. There was a significant positive correlation between NE in the brain and CORT in the serum (Figure 6F), although there was no significant correlation between NE in the brain and TNF-α in the serum, suggesting that the decrease in NE may also be involved in the inhibition of HPA axis hyperactivity.

## 5. Conclusions

**Figure 7:**
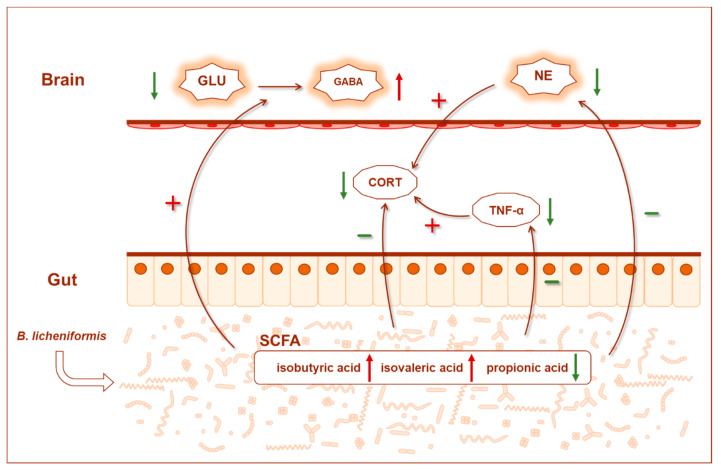
Graphical summary of the main findings. Effect of B. licheniformis on inhibiting HPA axis hyperactivity (reducing serum CORT) and inflammation (reducing serum TNF-α) by remodeling the gut microbiota and reducing anxiety (reducing anxiety-like behaviors and changing brain neurotransmitter levels) to alleviate the subhealth. The green arrow indicates a decrease, the red arrow indicates an increase, the green “−” indicates a negative effect, and the red “+” indicates a positive effect. GLU glutamic acid, GABA gamma-amino butyric acid, NE norepinephrine, CORT corticosterone, SCFAs short chain fatty acids.

In summary, short-term or potential abnormal physiological indices appeared in rats after subhealth treatment, and *B. licheniformis* intragastric administration for 4 weeks could alleviate the subhealth status in physiology and behavior. The possible mechanisms were as follows: *B. licheniformis* significantly reshaped the structural composition of the gut microbiota by increasing the degree of butyrate-producing bacteria in the microbial network, increasing the relative abundance of beneficial *Ruminococcaceae* and *Lachnospiraceae*, and decreasing the relative abundance of *Pasteurella*, which can lead to infectious diseases. On the one hand, the reshaped gut microbiota increased SCFAs levels in the colon, such as isobutyric acid and isovaleric acid, and reduced inflammation (such as reducing TNF-α). On the other hand, the gut microbiota may consume more propionic acid and then cause changes in the neurotransmitters in the brain, such as the decrease in Glu, increase in GABA and 5-HT, and so on. At the same time, it can help reduce NE in the brain, CORT and TNF-α in the serum, and then inhibit the hyperactivity of the HPA axis and reduce anxiety (Figure 7).

## Data Availability

All the 16S rDNA raw data reported in this paper have been deposited (PRJCA006102) in the Genome Sequence Archive (http://bigd.big.ac.cn/gsa, accessed on 31 August 2021) in the BIG Data Center, Chinese Academy of Sciences. Other published datasets used in this work have been listed in Supplementary information.

## Supplementary Materials

The following supporting information can be downloaded at: https://www.mdpi.com/article/10.3390/nu14081642/s1, Figure S1. Gut microbiota α-diversity (A) Richness index at week 4 (B) Chao1 index at week 4 (C) Shannon index at week 4 (D) Simpson index at week 4 (E) Richness index at week 8 (F) Chao1 index at week 8 (G) Shannon index at week 8 (H) Simpson index at week 4. * *p* < 0.05; ** *p* < 0.01; *** *p* < 0.001; **** *p* < 0.0001; Table S1: CUMS process; Table S2: gut microbiota network diagram.
