# Supplementary figures and images for: Bacillus licheniformis Reshapes the Gut Microbiota to Alleviate the Subhealth
Source: Nutrients. 2022 Apr 14;14(8):1642. doi: 10.3390/nu14081642 (PMC9025434; doi:10.3390/nu14081642)

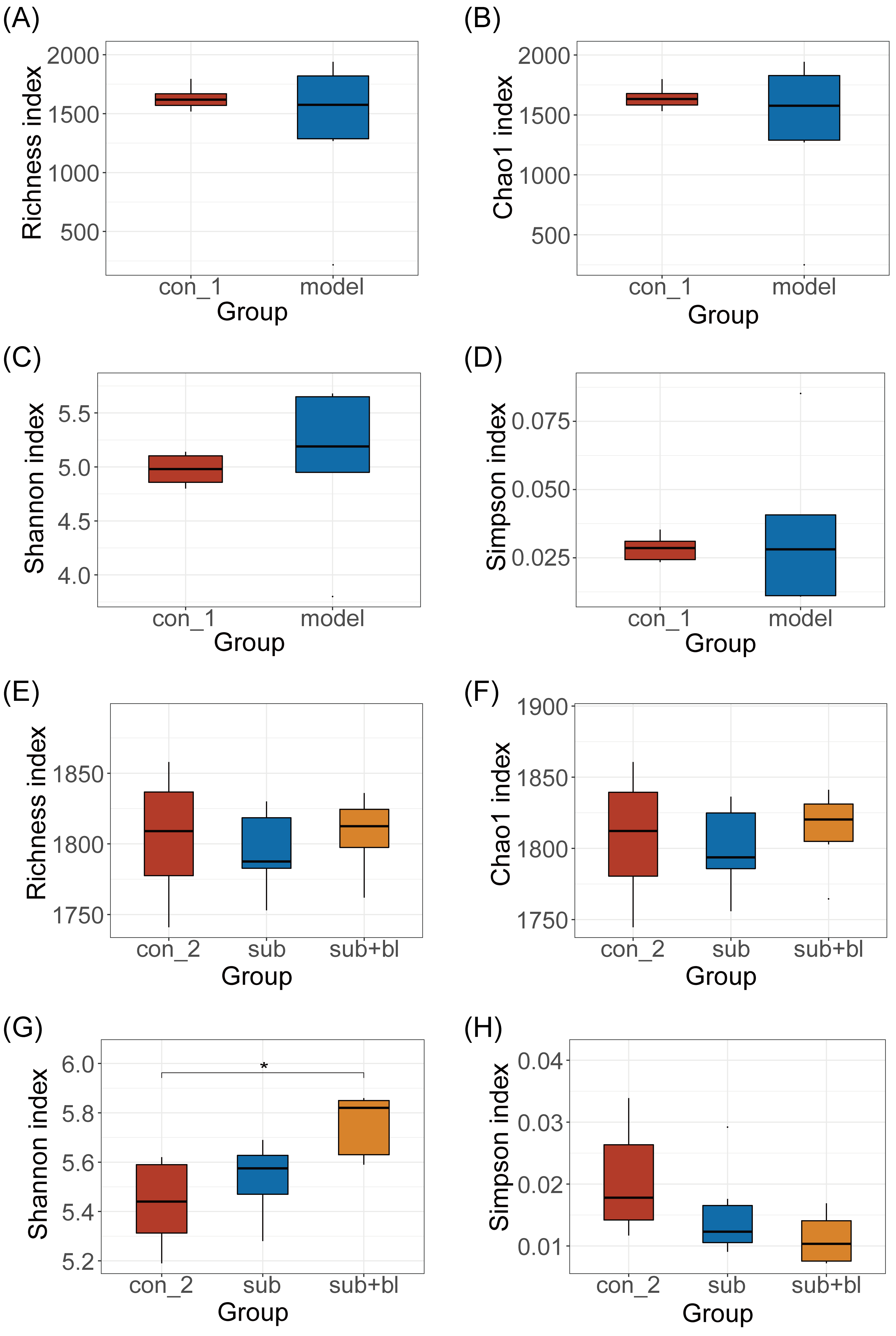

Supplement: Supplementary file 1 [file nutrients-14-01642-s001.zip › Figure S1.tif]
